# Factors Associated With Use of and Satisfaction With Telehealth by Adults in Rural Virginia During the COVID-19 Pandemic

**DOI:** 10.1001/jamanetworkopen.2021.19530

**Published:** 2021-08-05

**Authors:** Maria D. Thomson, Abigail C. Mariani, April R. Williams, Arnethea L. Sutton, Vanessa B. Sheppard

**Affiliations:** 1 Department of Health Behavior and Policy, Virginia Commonwealth University School of Medicine, Richmond; 2 Office of Community Outreach and Engagement, Massey Cancer Center, Richmond, Virginia

## Abstract

This survey study examines the use of and satisfaction with telehealth services by adults in rural Virginia during the COVID-19 pandemic.

## Introduction

COVID-19 has accelerated the expansion of telehealth, heralding an opportunity to integrate technology into clinical care delivery in new and purposeful ways. However, there are disparities among people in rural communities that limit opportunities to gain experience and comfort using technology for health information and services, including lower home broadband access, lower health literacy, and less use of online health information compared with urban populations.^1,2^ In this survey study, we examine the use of and satisfaction with telehealth services during the pandemic in a predominantly rural sample and estimate the magnitude of the association between demographic and health characteristics, health literacy, internet access, and the odds of using telehealth.

## Methods

### Sample

The Virginia Commonwealth University institutional review board approved this study, which follows the American Association for Public Opinion Research (AAPOR) reporting guideline. Respondents were recruited through the Virginia Living Well Registry (VALW), a community-based convenience sample registry of adults residing in primarily rural Virginia counties (Rural-Urban Continuum Codes 4-9). A total of 401 participants registered to the VALW before January 2020 were invited to complete a self-administered consent and survey online or through mailed paper surveys between June 2020 and January 2021. A waiver of signed consent was obtained for mailed surveys to enable survey completion via telephone. Additional information on study methods is available in the eAppendix in the Supplement. The overall response rate was 61%. An additional 6 participants who completed the VALW after June 2020 were included.

### Measures

Outcomes were self-reported telehealth use (yes vs no) and patient satisfaction with telehealth services^3^ since March 2020. Telehealth included communication via telephone, video, or electronic monitoring systems. Single-item screeners were used to identify low or inadequate health literacy,^4^ health insurance coverage, internet access, and overall perceived health. Health literacy was operationalized as perceived confidence completing medical forms independently, which has been shown to successfully identify individuals with low health literacy.^4^ Race and ethnicity were self-reported using categories defined by Office of Management and Budget standards. Race and ethnicity were analyzed in this study because disparities in preventive care use by race/ethnicity are well documented; in Virginia, rural Black residents experience greater incidence and/or mortality for some screenable cancers compared with White residents, suggesting that there are disparities in access to care. Rurality was categorized as Rural-Urban Continuum Codes 4 to 9 using participant address.^5^ Higher Perceived Stress Scale^6^ scores indicated greater perceived stress and were included given the pandemic context, which may be associated with health care seeking.

### Statistical Analysis

Means, SDs, frequencies, and proportions were used to describe demographic and health characteristics. Stratified analyses using 2-sided *t* tests and χ^2^ tests were used to examine potential differences between telehealth users vs nonusers, and satisfaction with telehealth. Multiple logistic regression was used to determine significance (*P* < .05) and magnitude of associations using SAS statistical software version 9.4 (SAS Institute).

## Results

The 253 participants (183 women [77.87%]) had a mean (SD) age, of 52.41 (16.12) years; 135 participants (57.69%) were non-Hispanic White and 157 (70.72%) lived in rural areas. Table 1 displays the full demographic characteristics of the participants. After March 2020, 102 participants (41.00%) reported telehealth use. Eighty participants (78.00%) were comfortable communicating with clinicians using telehealth, and 81 (79.00%) said they would use telehealth again. Some participants (69 participants [68.00%]) agreed that telehealth is an acceptable mode for health care delivery. Satisfaction among the 102 participants who used telehealth was associated with regular access to the internet (χ^2^_1_ = 4.58; *P* = .03) and higher health literacy (χ^2^_1_ = 5.02; *P* = .03) compared with those who were not satisfied. Table 2 displays the results of the multiple logistic regression. Factors significantly associated with higher odds of telehealth use included high health literacy (odds ratio, 2.93; 95% CI, 1.42-6.04) and perceived stress (adjusted odds ratio, 1.17; 95% CI, 1.05-1.31). No demographic differences were associated with telehealth satisfaction or use.

**Table 1.** Respondent Characteristics and Differences by Telehealth Use and Telehealth Satisfaction

Characteristic	Respondents, No. (%)				
	Total (N = 253)	Telehealth user (n = 102)	Nonuser (n = 149)	Satisfied with telehealth (n = 71)	Unsatisfied with telehealth (n = 31)
Age, mean (SD), y	52.41 (16.12)	50.93 (15.86)	53.27 (16.36)	50.12 (15.78)	53.00 (16.20)
Race/ethnicity^a^					
Non-Hispanic					
White	135 (57.69)	53 (55.79)	81 (59.12)	38 (57.58)	15 (51.72)
Black	90 (38.46)	40 (42.11)	49 (35.77)	26 (39.39)	14 (48.28)
Hispanic	3 (1.28)	2 (2.11)	1 (0.73)	2 (3.03)	0
American Indian	3 (1.28)	0	3 (2.19)	0	0
Asian	2 (0.85)	0	2 (1.46)	0	0
Unknown	1 (0.00)	0	1 (0.73)	0	0
Sex					
Female	183 (77.87)	77 (81.05)	105 (76.09)	56 (84.85)	21 (72.41)
Male	52 (22.13)	18 (18.95)	33 (23.91)	10 (15.15)	8 (27.59)
Reside in rural areas (Rural-Urban Continuum Codes 4-9)	157 (70.72)	57 (64.77)	98 (74.24)	39 (61.90)	18 (72.00)
Good overall health	201 (79.76)	77 (75.49)	122 (82.43)	54 (76.06)	23 (74.19)
Perceived Stress Scale score, mean (SD)	5.48 (3.20)	6.23 (3.50)	4.98 (2.89)	6.25 (3.40)	6.16 (3.78)
Health insurance					
Public insurance	66 (27.27)	32 (31.37)	34 (24.46)	21 (29.58)	11 (35.48)
Private insurance	135 (55.79)	58 (56.86)	76 (54.68)	43 (60.56)	15 (48.39)
Other	29 (11.98)	9 (8.82)	20 (14.39)	5 (16.13)	4 (5.63)
Uninsured	12 (4.96)	3 (2.94)	9 (6.47)	0	3 (4.23)
Regular access to internet	204 (88.31)	85 (89.47)	118 (88.06)	62 (93.94)	23 (79.31)
High health literacy	143 (64.41)	64 (73.56)	78 (58.65)	44 (75.86)	20 (68.97)

^a^ Racial/ethnic differences by telehealth use and satisfaction were tested as differences between non-Hispanic White and underrepresented racial and ethnic individuals (non-Hispanic Black, Hispanic, American Indian, Asian, and unknown).

**Table 2.** Logistic Regression Model of Factors Associated With Telehealth Use

Factor	OR (95% CI)	
	Unadjusted	Adjusted
Age	0.99 (0.96-1.01)	1.00 (0.98-1.02)
Underrepresented racial/ethnic group^a^	0.87 (0.51-1.48)	1.47 (0.75-2.89)
Female	1.34 (0.71-2.56)	1.02 (0.47-2.23)
Urban	0.64 (0.36-1.15)	1.21 (0.60-2.45)
No insurance	2.15 (0.57-8.15)	0.15 (0.02-1.31)
No internet	1.15 (0.50-2.66)	1.85 (0.65-5.23)
Perceived stress	1.13 (1.04-1.23)	1.17 (1.05-1.31)
High health literacy	1.96 (1.09-3.53)	2.93 (1.42-6.04)

Abbreviation: OR, odds ratio.

^a^ Because of the very small participant samples among Hispanic, American Indian, and Asian respondents, race/ethnicity was dichotomized as non-Hispanic White and underrepresented racial and ethnic groups (African American/Black, Hispanic, Asian, and American Indian).

## Discussion

Utilization of and satisfaction with telehealth services in this sample were associated with regular internet access, higher health literacy, and greater perceived stress. Demographic variables were not significantly associated with use of telehealth. Limitation of this study are that the convenience sample has implications for generalizability, we did not differentiate between modalities of telehealth use, and health literacy was measured with a 1-item screener; however, this screener has been shown to reliably differentiate high vs low health literacy.^6^ Implementation of telehealth will continue after the pandemic, and our work highlights key considerations for rural residents to ensure that existing technology barriers are not exacerbated.

## Notes

Supplement. **eAppendix.** Supplemental Methods eReferences
