# Supplementary material for: Factors Associated With Use of and Satisfaction With Telehealth by Adults in Rural Virginia During the COVID-19 Pandemic
Source: JAMA Netw Open. 2021 Aug 5;4(8):e2119530. doi: 10.1001/jamanetworkopen.2021.19530 (PMC8343464; doi:10.1001/jamanetworkopen.2021.19530)
Supplement: Supplement. — eAppendix. Supplemental Methods eReferences [file jamanetwopen-e2119530-s001.pdf]

Supplemental Online Content

Thomson MD, Mariani AC, Williams AR, Sutton AL, Sheppard VB. Factors associated with use of and satisfaction with telehealth by adults in rural Virginia during the COVID-19 pandemic. *JAMA Netw Open*. 2021;4(8):e2119530. doi:10.1001/jamanetworkopen.2021.19530

eAppendix. Supplemental Methods
eReferences

This supplemental material has been provided by the authors to give readers additional information about their work.

© 2021 Thomson MD et al. *JAMA Network Open.*

eAppendix. Supplemental Methods

Background and Overview of VLWR

Virginia Living Well Community Research Registry (VALW) is a community-based convenience sample registry of adults residing in primarily rural Virginia counties (RUCC 4-9). Racial and ethnic minorities as well as rural communities have documented underrepresentation in research and clinical trials.[1] The Community Outreach and Engagement (COE) office of the Massey Cancer Center created the VALW to help identify community needs and inform cancer and health programming priorities and to inform catchment specific research priorities for these priority populations. Our recruitment processes is guided by the TRUST model[2], which recommends the use of interdisciplinary teams, bilingual and bicultural staff, social media and social network focused recruitment methods, community partners, materials that are tailored for literacy, linguistic and cultural appropriateness. Massey Cancer Center has two satellite offices for Community Outreach and Engagement activities that are staffed by community members. These staff are multilingual and members of the community in which they work; they are able to leverage strong community networks to introduce VALW and invite participation. Similarly, snowball sampling and recruitment through social networks using word of mouth and social media and wider community networks (e.g., partnerships with local organizations, businesses, churches, community centers), radio advertising, comment card boxes in local businesses where people can request information is also being used. Recruitment is completed by the COE staff members throughout the catchment. VALW can be completed online using an entirely self-directed modality which includes e-consent or can be completed in-person using a paper/pencil or over the telephone (survey administered by the COE research staff) modality.

Study Measures

Outcomes

Telehealth Service Use. Telehealth was the primary outcome measure and was defined as "receiving healthcare from a provider over the phone, video conferencing, or through electronic monitoring systems." The survey question asked, "Have you used telehealth since the Governor's stay at home order?" Responses were dichotomized yes/no.

Experience with Telehealth Services. The overall satisfaction with telehealth services question from the Telehealth and Usability Questionnaire[3] was used to assess the extent to which those whose used any telehealth modality were satisfied or unsatisfied with their experience. This question used a 5-point Likert scale. Responses were dichotomized as yes ("agree", "strongly agree") vs. no ("neutral", "disagree", "strongly disagree"). Neutral responses were grouped as disagreement to compare those who specifically rated their experience as positive.

Demographics

Respondent Demographic and Health Characteristics. Demographic variables included age, race/ethnicity, sex, geographic location of residence, and single items for health insurance coverage, access to the internet and overall perceived health. Race and ethnicity was self-reported using provided categories recommended by OBM as standards for collection of race and ethnicity data.[4] Because of very small participant samples among Hispanic/Latinx, American Indian and Asian respondents, race and ethnicity was dichotomized to Non-Hispanic white and underrepresented racial/ethnic minorities (African American/Black, Hispanic, Asian, American Indian) for statistical analyses. Geographic location was determined using respondent's address and

© 2021 Thomson MD et al. *JAMA Network Open.*

associated RUCC classification; RUCC 4-9 are considered rural based on population size.[5] A four-item version of the Perceived Stress Scale was used to measure respondents' perceived stress.[6,7] Respondents rate how often they experienced stressful situations in the past month on a 5-point Likert scale ranging from "never" to "very often". Scores for respondents that missed one question were imputed as the mean of the remaining three questions; if more than one question was missing, the respondent score was removed from analysis. Total scores indicated greater levels of perceived stress. Overall health was measured with a single item question that is widely used; "In general, would you say your health is" and answers were collected using a 5-point Likert scale ranging from poo-excellent.

Independent Variables

Health Literacy. A single item health literacy screening question was used,[8] "How confident are you filling out medical forms by yourself?" This measure was validated using existing measures of health literacy, shown to perform better than other screening tools, as well as across a variety of demographics including age, race, ethnicity, language, and education.[9] This item used a 5-point Likert scale from "extremely confident" to "not at all confident." Responses were dichotomized following published instructions with scores of 3 or higher indicating low health literacy.[10]

Internet access. Participants were asked a single question (yes/no) about their access to regular internet with the following response options: Yes, I have regular access at home, Yes, I have regular access at work or another place, No, I do not have regular access

AAPOR Survey Disclosure Checklist

**Survey sponsor**	VCU Office of the Vice President for Research and Innovation and C. Kenneth and Dianne Wright Center for Clinical and Translational Research [unnumbered]
**Survey/Data collection supplier**	Manuscript authors listed on paper
**Population represented**	Adults in Virginia state, United States
**Sample size**	253
**Mode of data collection**	Online and traditional mail (paper)
**Type of sample (probability/non-probability)**	Non-probability
**Start and end dates of data collection**	June-January 2021
**Are the data weighted?**	No
**Contact for more information**	Dr. Maria Thomson, Virginia Commonwealth University, maria.thomson@vcuhealth.org

© 2021 Thomson MD et al. *JAMA Network Open.*

Survey Response Rates

Adults living in Virginia who had completed the VALW registry prior to January 2020 (n=401) and new registry participants (n=6) after June 2020 (total n=407) were invited to complete a self-administered online survey or paper survey between June 2020 and January 2021. Only the original 401 VALW registry participants who were recontacted via the registry were used to calculate the response rate. Of those 401, a total of 247 surveys were completed by January 2021; all invitees were known to be eligible, 145 refused to respond, 8 invitations were not deliverable, and 1 registry participant was deceased at the time the invitation was sent. Using the American Association of Public Opinion Research's Response Rate Calculator,[11] the survey response rate was 61.7% and the survey cooperation rate was 62.9%.

eREFERENCES

1. Yabroff KR, Han X, Zhao J, Nogueira L, Jemal A. Rural Cancer Disparities in the United States: A Multilevel Framework to Improve Access to Care and Patient Outcomes. *JCO Oncol Pract.* 2020;16(7):409-413. doi:10.1200/op.20.00352
2. Sheppard VB, Cox LS, Kanamori MJ, et al. Brief report: If you build it, they will come methods for recruiting Latinos into cancer research. *J Gen Intern Med.* 2005;20(5):444-447. doi:10.1111/j.1525-1497.2005.0083.x
3. Parmanto B, Lewis, Jr. AN, Graham KM, Bertolet MH. Development of the Telehealth Usability Questionnaire (TUQ). *Int J Telerehabilitation.* 2016;8(1):3-10. doi:10.5195/ijt.2016.6196
4. U.S. Department of Health and Human Services. Implementation guidance on data collection standards for race, ethnicity, sex, primary language, and disability status. Published online 2011:1-10.
5. USDA ERS - Rural-Urban Continuum Codes. Accessed October 23, 2019. https://www.ers.usda.gov/data-products/rural-urban-continuum-codes/
6. Warttig SL, Forshaw MJ, South J, White AK. New, normative, English-sample data for the Short Form Perceived Stress Scale (PSS-4). *J Health Psychol.* 2013;18(12):1617-1628. doi:10.1177/1359105313508346
7. Cohen S, Kamarck T, Mermelstein R. A Global Measure of Perceived Stress. *J Health Soc Behav.* 1983;24(4):385. doi:10.2307/2136404
8. Chew LD, Bradley KA, Boyko EJ. Brief questions to identify patients with inadequate health literacy. *Fam Med.* 2004;36(8):588-594.
9. Sarkar U, Karter AJ, Liu JY, Moffet HH, Adler NE, Schillinger D. Hypoglycemia is more common among type 2 diabetes patients with limited health literacy: The diabetes study of northern California (distance). *J Gen Intern Med.* 2010;25(9):962-968. doi:10.1007/s11606-010-1389-7
10. Chew LD, Griffin JM, Partin MR, et al. Validation of screening questions for limited health literacy in a large VA outpatient population. *J Gen Intern Med.* 2008;23(5):561-566. doi:10.1007/s11606-008-0520-5
11. Response Rates - An Overview - AAPOR. https://www.aapor.org/Education-Resources/For-Researchers/Poll-Survey-FAQ/Response-Rates-An-Overview.aspx

© 2021 Thomson MD et al. *JAMA Network Open.*
